# Supplementary material for: Modeling glioblastoma heterogeneity as a dynamic network of cell states
Source: Mol Syst Biol. 2021 Sep 16;17(9):e10105. doi: 10.15252/msb.202010105 (PMC8444284; doi:10.15252/msb.202010105)
Supplement: Supplementary file 5 — Source Data for Figure 3 [file MSB-17-e10105-s001.zip › Figure3A_sourcedata/GSEA_3065/hallmarks_state3.GseaPreranked.1623416274744/HALLMARK_MYC_TARGETS_V2.html]

Details for gene set HALLMARK\_MYC\_TARGETS\_V2[GSEA]

|  || Dataset | state3 |
| Phenotype | NoPhenotypeAvailable |
| Upregulated in class | na\_neg |
| GeneSet | HALLMARK\_MYC\_TARGETS\_V2 |
| Enrichment Score (ES) | -0.19847733 |
| Normalized Enrichment Score (NES) | -0.61705965 |
| Nominal p-value | 0.9595469 |
| FDR q-value | 0.9894597 |
| FWER p-Value | 1.0 |
Table: GSEA Results Summary

  

Fig 1: Enrichment plot: HALLMARK\_MYC\_TARGETS\_V2      
 Profile of the Running ES Score & Positions of GeneSet Members on the Rank Ordered List

  

| PROBE | GENE SYMBOL | GENE\_TITLE | RANK IN GENE LIST | RANK METRIC SCORE | RUNNING ES | CORE ENRICHMENT || 1 | DDX18 |  |  | 279 | 0.250 | 0.0356 | No |
| 2 | MCM4 |  |  | 482 | 0.201 | 0.0667 | No |
| 3 | TMEM97 |  |  | 740 | 0.165 | 0.0818 | No |
| 4 | NOP56 |  |  | 829 | 0.155 | 0.1135 | No |
| 5 | UTP20 |  |  | 1002 | 0.138 | 0.1311 | No |
| 6 | SLC19A1 |  |  | 1494 | 0.103 | 0.1026 | No |
| 7 | MPHOSPH10 |  |  | 1578 | 0.099 | 0.1198 | No |
| 8 | MYBBP1A |  |  | 1686 | 0.093 | 0.1325 | No |
| 9 | TCOF1 |  |  | 1847 | 0.084 | 0.1368 | No |
| 10 | MYC |  |  | 2324 | 0.064 | 0.0993 | No |
| 11 | MCM5 |  |  | 2344 | 0.063 | 0.1141 | No |
| 12 | LAS1L |  |  | 2472 | 0.058 | 0.1152 | No |
| 13 | NPM1 |  |  | 2533 | 0.056 | 0.1234 | No |
| 14 | HSPD1 |  |  | 2700 | 0.050 | 0.1178 | No |
| 15 | MRTO4 |  |  | 2921 | 0.043 | 0.1042 | No |
| 16 | PPAN |  |  | 2927 | 0.043 | 0.1153 | No |
| 17 | PPRC1 |  |  | 3412 | 0.030 | 0.0679 | No |
| 18 | GNL3 |  |  | 4103 | 0.012 | -0.0080 | No |
| 19 | FARSA |  |  | 4127 | 0.012 | -0.0075 | No |
| 20 | PA2G4 |  |  | 4363 | 0.005 | -0.0332 | No |
| 21 | PRMT3 |  |  | 4529 | 0.001 | -0.0520 | No |
| 22 | GRWD1 |  |  | 4700 | -0.004 | -0.0705 | No |
| 23 | UNG |  |  | 4979 | -0.010 | -0.0997 | No |
| 24 | PLK1 |  |  | 5235 | -0.017 | -0.1245 | No |
| 25 | WDR43 |  |  | 5243 | -0.017 | -0.1208 | No |
| 26 | NIP7 |  |  | 5297 | -0.018 | -0.1219 | No |
| 27 | PES1 |  |  | 5501 | -0.024 | -0.1387 | No |
| 28 | IMP4 |  |  | 5520 | -0.025 | -0.1341 | No |
| 29 | RRP9 |  |  | 5569 | -0.026 | -0.1325 | No |
| 30 | WDR74 |  |  | 5575 | -0.026 | -0.1260 | No |
| 31 | SUPV3L1 |  |  | 5585 | -0.027 | -0.1198 | No |
| 32 | BYSL |  |  | 5770 | -0.032 | -0.1323 | No |
| 33 | PHB |  |  | 5852 | -0.034 | -0.1323 | No |
| 34 | RABEPK |  |  | 5926 | -0.037 | -0.1308 | No |
| 35 | NOLC1 |  |  | 6241 | -0.048 | -0.1539 | No |
| 36 | AIMP2 |  |  | 6394 | -0.052 | -0.1572 | No |
| 37 | NOC4L |  |  | 6449 | -0.054 | -0.1487 | No |
| 38 | EXOSC5 |  |  | 6616 | -0.062 | -0.1511 | No |
| 39 | CBX3 |  |  | 7029 | -0.083 | -0.1761 | Yes |
| 40 | TBRG4 |  |  | 7225 | -0.095 | -0.1727 | Yes |
| 41 | NOP16 |  |  | 7350 | -0.104 | -0.1588 | Yes |
| 42 | NDUFAF4 |  |  | 7552 | -0.122 | -0.1489 | Yes |
| 43 | DCTPP1 |  |  | 7845 | -0.158 | -0.1398 | Yes |
| 44 | HSPE1 |  |  | 7918 | -0.170 | -0.1021 | Yes |
| 45 | CDK4 |  |  | 8168 | -0.220 | -0.0713 | Yes |
| 46 | SRM |  |  | 8693 | -0.511 | 0.0067 | Yes |
Table: GSEA details [plain text format]

  

Fig 2: HALLMARK\_MYC\_TARGETS\_V2: Random ES distribution      
 Gene set null distribution of ES for **HALLMARK\_MYC\_TARGETS\_V2**
